# Coronary-Pulmonary Artery Fistula Repair With Coil Embolization: A Single Center Experience

**DOI:** 10.7759/cureus.28407

**Published:** 2022-08-25

**Authors:** Imran Sulemankhil, Ahmed H Mohamed, Syed A Gilani

**Affiliations:** 1 Department of Cardiovascular Medicine, University of Texas Medical Branch at Galveston, Galveston, USA

**Keywords:** coil embolization, left anterior descending coronary artery to pulmonary artery fistula, trans-catheter closure, coronary-pulmonary artery fistulas, coronary artery fistula

## Abstract

Coronary-pulmonary artery fistulas (CPF) are a rare malformation that is often asymptomatic but can be associated with dyspnea, angina, palpitation, dizziness, and syncope. Trans-catheter closure (TCC) with coil embolization is gaining prominence relative to surgical closure due to lower complications; however, there is a paucity of literature on the closure of CPFs with TCC. Here, we demonstrate a case series on the closure of a left anterior descending (LAD) artery to pulmonary artery (PA) fistula by advancing a guideliner into the coronary artery up to the origin of the coronary fistula in order to provide support for the advancement of the microcatheter and coil delivery.

## Introduction

Coronary artery fistula (CAF) is a rare congenital or acquired cardiac abnormality where the coronary artery bypasses the myocardial capillaries and communicates directly with either the cardiac chamber or one of the great vessels [1]. This rare malformation was previously estimated to occur in 0.1-0.2% of the population using invasive angiography, but with computed tomographic (CT) angiography it has been estimated to be present in 0.9% of the population [2]. Coronary-pulmonary artery fistulas (CPF) are a subtype of CAFs that account for 15-30% of CAF cases and refer to abnormal communication between the coronary artery and pulmonary artery (PA) [3]. The embryological origins of CPFs are proposed by the Hackensellner involution-persistence hypothesis. It is suspected that rather than involution of the four truncus branches, there is the persistence of the pulmonary sinus branch which makes an abnormal connection with the aortic sinus coronary arteries [4].

Although most CPFs are asymptomatic, symptoms including dyspnea, angina, palpitation, dizziness, and syncope are more commonly noted with larger diameter fistulas, multiple fistulas, and severe left-to-right shunts [5]. The 2018 American College of Cardiology/American Heart Association Task Force guidelines recommend a thorough review by an interdisciplinary team of congenital or non-congenital cardiologists and surgeons to determine the role of percutaneous or surgical closure [6]. As there is low morbidity, recommendations are based on a small case series and the choice of management is patient-specific. Trans-catheter closure (TCC) with coil embolization is the preferred intervention, but surgical ligation may be favored for patients with multiple drainage sites, distal fistula origin, multiple fistulas or large branch vessels, and/or concomitant cardiac disorders [7].

As there is a paucity of CPF cases treated with TCC, we present a case series of three cases of patients with the left anterior descending (LAD) coronary artery to PA fistulas that were successfully closed with coil embolization.

## Case presentation

Case 1

**Figure 1:**
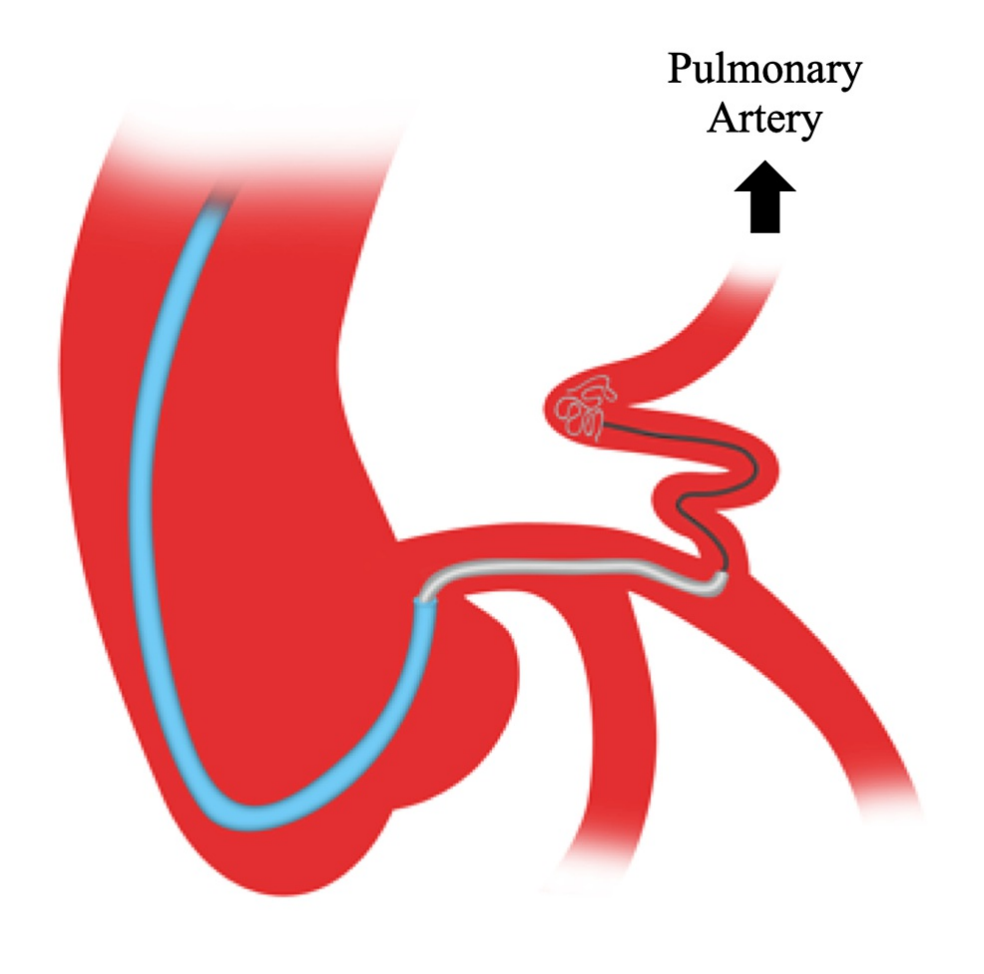
A guide catheter (blue) was used to engage the left main coronary artery. Using a telescoping technique, the guideliner (gray) was advanced over the guidewire up to the origin of the left anterior descending artery to pulmonary artery fistula. With the guideliner providing support, a microcatheter wire (black) was advanced over the guidewire into the fistula, followed by coil delivery.

**Figure 2:**
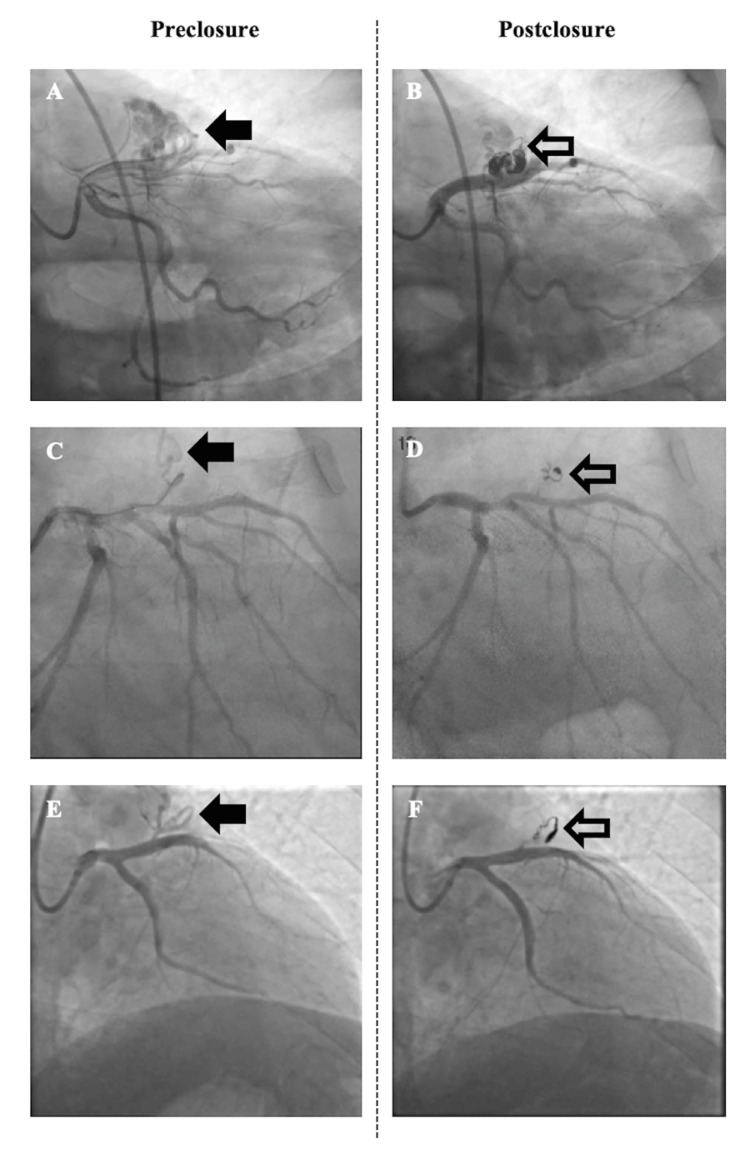
Pre and post closure coronary angiography images, respectively, of (A, B) case 1, (C, D) case 2, and (E, F) case 3. Left anterior descending coronary artery-to-pulmonary artery fistula (solid arrow) and coil (open arrow).

A 65-year-old male with a medical history significant for hypertension, chronic kidney disease stage III, diet-controlled diabetes mellitus (DM), bicuspid aortic valve, and mild aortic root aneurysm was seen in the cardiology clinic for evaluation of chronic dyspnea on exertion which was more pronounced for the past two years. The patient had noticed a lifelong history of limited exertional capacity and dyspnea on exertion, which had been attributed to allergies and asthma. An electrocardiogram (EKG) showed normal sinus rhythm with no Q waves or repolarization abnormalities. The echocardiography noted a normal left ventricular ejection fraction but was significant for mild to moderate aortic stenosis (AVA 1.2 cm^2^ and an AV mean gradient of 16 mmHg). Computed tomography angiography was significant for a 46 mm fusiform ascending aortic aneurysm and a LAD to PA fistula. The decision was made to pursue right and left heart catheterization, which did not show any obstructive coronary artery disease (CAD) but did note a large and tortuous mid-LAD to pulmonary artery fistula. The LAD proximal to the fistula origin was enlarged due to increased flow from the fistula. Pulmonary artery pressures were normal and no significant step up in oxygen saturation was noted in the proximal right and left pulmonary arteries. As there was a continuous shunting flow resulting in coronary steal, this was likely the etiology of the dyspnea on exertion. The decision was made to pursue percutaneous coil embolization (Figure 1). Using a right common femoral artery access, a 7 Fr EBU 3.5 guide catheter (Medtronic, Inc., Minneapolis, MN) was advanced to the left main coronary artery and a Balance Middleweight (BMW) Universal 0.014’’ wire (Abbott Vascular, Chicago, IL) was passed into the fistula. Then a 7Fr guideliner was advanced over the BMW wire into the LAD up to the origin of the fistula as it provided support for the advancement of the microcatheter and coil delivery. This was followed by advancing the Renegade 0.021’’ microcatheter (Boston Scientific, Boston, MA) over the BMW wire deep into the fistula. The BMW wire was then removed, and multiple interlock coils (5 × 8, 4 × 15, 5 × 15, 6 × 20, and 8 × 20) were deployed with good occlusion of the main fistula vessel (Figure 2A-2B). A residual proximal small side feeder vessel was noted, which was engaged using the BMW wire and microcatheter followed by successful closure with a 3 × 6 Interlock coil. A repeat angiogram did not show residual flow from the LAD to the PA and there was complete occlusion of the LAD to PA fistula. On follow-up two months later, the patient had complete resolution of his dyspnea on exertion.

Case 2

A 70-year-old male with a medical history significant for morbid obesity (BMI 44), sedentary lifestyle, hypertension, uncontrolled DM II, dermatomyositis, and obstructive sleep apnea (untreated) was admitted to the hospital for gradually worsening shortness of breath on minimal exertion and exertional fatigue. During hospitalization, the patient was diagnosed with new onset acute systolic heart failure (LVEF 30-35%), atrial fibrillation, and a non-ST-elevation myocardial infarction. He underwent heart catheterization that showed a complex three-vessel CAD and a proximal LAD to PA fistula. He was treated with aggressive heart failure therapy and, after a detailed heart team evaluation, he was referred for a percutaneous coronary intervention (PCI) for coronary revascularization. The patient underwent PCI of the LAD, circumflex, and right coronary artery with an impella support device. He was discharged home and brought back electively for the LAD to PA fistula closure to prevent coronary steal, improve the LAD territory ischemia, and help improve the residual dyspnea on exertion symptoms. For the fistula closure procedure, a 7Fr right common femoral access was obtained. A 7Fr EBU 4.0 Launcher guide catheter (Medtronic, Inc., Minneapolis, MN) was advanced to the left main coronary artery. Then a choice PT wire (Boston Scientific, Boston, MA) was advanced to the LAD to PA fistula, followed by advancement of a 7Fr guideliner over the choice PT wire up to the LAD fistula origin to provide support for the guidewire/microcatheter advancement and coil delivery (Figure 1). The choice PT wire was further advanced into the fistula and the Renegade 0.021’’ microcatheter was then advanced deep into the fistula past the first two bends (proximal to any major fistula branches). Upon removal of the guidewire, a 3 × 6 Interlock coil was deployed with good occlusion of the fistula and a repeat angiogram did not show residual flow (Figure 2C-2D). The patient was discharged in stable condition with aspirin and clopidogrel for his prior CAD/PCIs. The patient had gradual improvement in his exercise tolerance and dyspnea on exertion symptoms at a five-month follow-up.

Case 3

A 53-year-old male with a past medical history significant for hypertension and excessive premature ventricular contractions (PVCs) presented with worsening exertional dyspnea over several months. He underwent an exercise stress echocardiogram that showed anteroseptal ischemia and reduced exercise capacity. Coronary angiography did not show any obstructive CAD but was significant for a large proximal LAD to PA fistula. The right heart and pulmonary pressures were normal and no significant step up in oxygen saturation was noted in the right or left PA. LAD to PA fistula-related coronary steal was considered to be the likely etiology of the anterior ischemia and exertional symptoms. After evaluation by the heart team, the decision was made to pursue percutaneous coil embolization (Figure 1). Using right common femoral artery access, a 7Fr EBU 3.5 guide catheter was engaged in the left main coronary artery. A BMW 0.014’’ universal wire was advanced to the LAD and a 7Fr guideliner was placed in the left main coronary artery for support. The BMW wire was removed and, using a Choice PT 0.014" wire, a 0.021" Progreat microcatheter (Terumo Medical Corporation, Somerset, New Jersey, USA) was advanced into the fistula. The Choice PT wire was removed and a 4 × 13 AzurCx coil was successfully released into the fistula (Figure 2E-2F). A repeat coronary angiogram showed successful closure of the fistula with no further flow. However, despite successful closure of the LAD to PA fistula, the patient continued to have some residual shortness of breath and palpitations that was related to excessive PVCs.

## Discussion

Coronary-pulmonary artery fistulas are a very rare entity, and the majority of CPFs arise from the left anterior descending or right coronary artery and drain into the main pulmonary trunk via single or multiple small-caliber fistulas [8,9]. Although most patients with CPFs are asymptomatic, the size and number of fistulas may result in hemodynamic disturbance-related symptoms such as dyspnea, angina, palpitation, dizziness, and syncope. Moreover, there is an increased risk of complications such as myocardial ischemia, heart failure, infective endocarditis, arrhythmias, pulmonary hypertension, presence of aneurysms, and CAF rupture or thrombosis [5,10]. These are more common with single large fistulas; however, multiple fistulas may also precipitate symptoms with age, as was noted in the patient in case 1. Hence, appropriate management may be to occlude the dominant fistula [1].

Due to the paucity of literature regarding these abnormalities, there has not been a consensus by the American College of Cardiology/American Heart Association regarding their management with TCC or surgical intervention. Although surgical ligation has been noted to result in reduced late survival due to perioperative myocardial infarction or high rates of residual tricuspid valve regurgitation, it is favored when the following factors are present: multiple drainage sites, distal fistula origin, multiple fistulas or large branch vessels, tortuosity of the vessel, and/or concomitant cardiac disorders [7,11]. TCC, on the other hand, is less invasive and has fewer iatrogenic complications. However, it should be noted that coil embolization in patients with large fistulas or high-flow shunts can result in complications such as residual leakage, recanalization of the fistulas, coil migration, and distal embolization [5]. To mitigate these events, vessel anatomy can be used to guide optimal coil size selection and location of coil deployment. Whereas an ideal coil size should be greater than 10-20% of the artery, the deployment of the coil should be beyond the first or second curve of the fistula, as was achieved in our cases [5]. Furthermore, this case series highlights using a guide catheter to approximate the left main artery, then using a guideliner to intubate the LAD up to the fistula origin to support the guidewire, microcatheter, and coil embolization. This approach provided a safe alternative to deep seating the guide catheter for support during coil embolization (Figure 1). In addition to providing support, advancing the guideliner up to the origin of the coronary fistula prevented the microcatheter from prolapsing into the proximal coronary artery, which had already enlarged due to chronic increased fistula-related flow, during the advancement of the coils into the fistula.

## Conclusions

In this case series, we describe three patients with LAD to PA fistulas who had successful coil embolization. The procedures were well tolerated with intermittent improvement of their symptoms. This case series adds to the scarce literature on CPF intervention and highlights the technique of advancing a guideliner into the coronary artery up to the origin of the coronary fistula to provide support for the advancement of the microcatheter and coil delivery.

## References

[1] Yun G, Nam TH, Chun EJ (2021). Coronary artery fistulas: pathophysiology, imaging findings, and management. Radiographics.

[2] Said SA, el Gamal MI, van der Werf T (1997). Coronary arteriovenous fistulas: collective review and management of six new cases--changing etiology, presentation, and treatment strategy. Clin Cardiol.

[3] Kim MS, Jung JI, Chun HJ (2010). Coronary to pulmonary artery fistula: morphologic features at multidetector CT. Int J Cardiovasc Imaging.

[4] Heifetz SA, Robinowitz M, Mueller KH, Virmani R (1986). Total anomalous origin of the coronary arteries from the pulmonary artery. Pediatr Cardiol.

[5] Liu X, Zhang L, Qi Z, Fan M, Ge J (2019). The characteristics of coronary-pulmonary artery fistulas and the effectivity of trans-catheter closure: a single center experience. J Thorac Dis.

[6] Stout KK, Daniels CJ, Aboulhosn JA (2019). 2018 AHA/ACC guideline for the management of adults with congenital heart disease: a report of the American College of Cardiology/American Heart Association Task Force on clinical practice guidelines. Circulation.

[7] Kabbani Z, Garcia-Nielsen L, Lozano ML, Febles T, Febles-Bethencourt L, Castro A (2008). Coil embolization of coronary artery fistulas. A single-centre experience. Cardiovasc Revasc Med.

[8] Verdini D, Vargas D, Kuo A (2016). Coronary-pulmonary artery fistulas: a systematic review. J Thorac Imaging.

[9] Umaña E, Massey CV, Painter JA (2002). Myocardial ischemia secondary to a large coronary-pulmonary fistula--a case report. Angiology.

[10] Vitarelli A, De Curtis G, Conde Y (2002). Assessment of congenital coronary artery fistulas by transesophageal color Doppler echocardiography. Am J Med.

[11] Said SM, Burkhart HM, Schaff HV (2013). Late outcome of repair of congenital coronary artery fistulas--a word of caution. J Thorac Cardiovasc Surg.

